# Peripheral tuberculin purified protein derivative specific T cell immunoreactivity dynamics in non-muscle invasive bladder cancer patients receiving bacillus Calmette-Guerin instillation treatment

**DOI:** 10.3389/fonc.2022.927410

**Published:** 2022-10-27

**Authors:** Huangqi Duan, Weimin Xia, Ding Xu, Yingying Chen, Yu Ding, Chen Wang, Ruiming Sun, Chengcheng Yao, Shun Zhang, Yu Wu, Ping Ji, Shujun Wang, Subo Qian, Ying Wang, Haibo Shen

**Affiliations:** ^1^ Department of Urology, Xinhua Hospital, School of Medicine, Shanghai Jiao Tong University, Shanghai, China; ^2^ Shanghai Institute of Immunology, Department of Immunology and Microbiology, Shanghai Jiao Tong University School of Medicine, Shanghai, China

**Keywords:** non-muscle invasive bladder cancer (NMIBC), bacillus Calmette-Guerin, peripheral immune responses, cytokine profiles, predictor

## Abstract

Intravesical bacillus Calmette-Guerin (BCG) instillation is recommended as an adjuvant therapy for intermediate-risk and high-risk non-muscle invasive bladder cancer (NMIBC) after transurethral resection of bladder tumor (TURBt) with nearly 70% reoccurrence. In the present study, we investigated the dynamics of peripheral purified protein derivative (PPD)-specific immune responses along the treatment. Intravesical BCG instillation caused a significant increase in peripheral PPD-specific IFN-γ release of NMIBC patients, when compared to those receiving chemo-drug instillation. Through a follow-up study, we detected rapid increase in PPD-specific IFN-γ, IL-2, and IL-17A producing CD4^+^ and CD8^+^ T cells in the induction phase. Interestingly, the frequencies of PPD-specific IFN-γ and IL-2 producing CD4^+^ and CD8^+^ T cells decreased dramatically after induction treatment and were restored after BCG re-instillation, whereas IL-17A-producing T cells remained at the maintenance phase. However, we only observed that the percentages of peripheral CD8^+^ T cells were significantly higher in BCG responder patients than those in BCG refractory patients at the baseline with the potential of predicting the recurrence. A more dramatic increase in PPD-specific IFN-γ and IL-2 producing CD4^+^ and CD8^+^ T cells after one and two dose BCG instillations was observed in refractory NMIBC patients. Therefore, regional BCG instillation induced transient peripheral PPD-specific T cell responses, which could be restored through repetitive BCG instillation. Higher proportions of peripheral CD8^+^ T cells at baseline were associated with better responses to BCG instillation for the prevention of recurrence of bladder cancer.

## Introduction

Bladder cancer (BC) is the second most common malignancy in the urinary system. In China, 91,893 new cases and 42,973 deaths from BC were reported in 2022 (1). Approximately 70%−75% of BC patients are diagnosed with non-muscle-invasive bladder cancer (NMIBC) and undergo transurethral resection of bladder tumor (TURBt) (2, 3). Adjuvant intravesical therapy is recommended to reduce local recurrence and decrease the risk of disease progression (4). Chemotherapeutics and bacillus Calmette-Guerin (BCG) instillation are two of the most widely used regimens in the clinic. They exhibit clinical efficacy in decreasing the likelihood of BC relapse. BCG instillation was first introduced by Morales et al. in 1976 (5) and is presently the standard adjuvant therapy for intermediate risk and high risk NMIBC (6, 7). However, repetitive and high dose intravesical BCG instillation causes multiple side effects, such as frequent urination and dysuria in 30%−50% of the patients, as well as systemic toxicity including hyperpyrexia, hematuria, and hepatitis in 10%−20% of patients (8), often leading to the discontinuation of treatment. Furthermore, 30% of NMIBC patients fail to benefit from intravesical BCG instillation (7), showing a lack of predictive indicators for the prognosis of BCG instillation in NMIBC patients.

BCG is an attenuated strain of *Mycobacterium bovis* (9). BCG instillation is one of the most successful forms of cancer immunotherapy. When instilled into the bladder, live BCG attach to the cell membrane and are internalized by epithelial cells to induce local immune responses (7, 10). T cells can be detected in the urine of patients after the treatment, among which CD4^+^ T cells are predominant (11–14). Studies using mouse models indicated that the infiltration of immune cells into the bladder mucosa, particularly CD4^+^ and CD8^+^ T cells, was essential for eliminating the tumors (15, 16). In addition to local responses, BCG therapy also induces systemic immune responses. After receiving BCG instillation, more than 40% of patients become tuberculin skin test positive (17). It was reported that pre-existing BCG-specific T cells were beneficial for intravesical BCG instillation therapy, largely because they were more sensitized BCG-specific lymphocytes (18). The baseline cytokine profiles of PPD-specific CD4^+^ T cells in NMIBC are thought to predict the outcomes of BCG instillation (19).

In China, neonatal BCG immunization has been implemented since 1956 (20). Intravesical BCG instillation after TURBt provides an opportunity to re-encounter BCG. Whether BCG instillation induces systemic immune responses and the relationship with disease progression have not been addressed. In the present study, we investigated the dynamics of systemic immune responses after BCG therapy in both the induction and maintenance phases. Our findings provided insight into the possible mechanisms involved in the response to BCG instillation and were intended to identify potential immune indicators associated with clinical outcomes.

## Materials and methods

### Study subjects

In total 65 NMIBC patients were recruited in this study from the Department of Urology at Xinhua Hospital affiliated to Shanghai Jiao Tong University School of Medicine (**Table 1**). All of the participants were initially diagnosed with intermediate-risk or high-risk NMIBC based on the results of the biopsies from cystoscopy examination or TURBt surgery. Among them, 11 patients underwent at least eight treatments with chemotherapy using Epirubicin (defined as the “NON-BCG” group), and all other patients received intravesical BCG instillation therapy (120 mg/dose) (Rongsheng, Chengdu, China). BCG instillation therapy included six consecutive instillations each week (induction phase) followed by three weekly instillations at months 3, 6, 12, 18, 24, 30, and 36 (maintenance phase) (4). Cystoscopy and biopsies were conducted under regional anesthesia 6 weeks after each treatment phase. Of the patients, 17 patients received intravesical BCG instillation therapy at the maintenance phase (defined as the “BCG” group) and the remaining 37 patients were recruited for a follow-up study. Based on the results from cystoscopy and biopsies, 27 patients who showed no recurrence at 6 months from the beginning of the treatment were defined as “BCG responder”, and 10 underwent the recurrence at 6 months that were defined as “BCG refractory” (21). All patients signed informed consent forms. This study was approved by the EthicalCommittee of Xinhua Hospital, and the procedures performed in this study were in accordance with the 1964 Helsinki declaration.

**Table 1:** Characteristics of bladder cancer patients.

	Chemotherapy	BCG therapy			
	n=11	n=17*	n=37**		
			BCG responder n=27	BCG refractory n=10	*P* value
Age	68.81 ± 7.30	67.82 ± 7.73	68 ± 11.14	70 ± 11.00	NS
Gender					
Male	8	14	22	8	NS
Female	3	3	7	2	NS
TURBt Stage					
HgTa	5	8	11	5	NS
CIS	2	3	3	1	NS
T1	4	6	13	4	NS

*: BC patients at the maintenance stage **: BC patients in the follow-up study.

NS, no significance.

### PBMCs preparation and *in vitro* stimulation

Whole blood was collected into tubes containing ethylene diamine tetraacetic acid (EDTA) (ZHIYUAN, Wuhan, China) before BCG instillation therapy. Peripheral blood mononuclear cells (PBMCs) were isolated by Ficoll-Hypaque density gradient centrifugation with Lymphoprep^™^ solution (AXIS-SHIELD Poc AS, Oslo, Norway) according to the manufacturer’s recommendation. PBMCs were washed twice and resuspended in RPMI 1640 complete medium containing 10% fetal calf serum (FCS), and 1% penicillin and streptomycin (Gibco^™^, Invitrogen Corporation, USA) at a concentration of 7.5 × 10^6^ per mL. PBMCs were incubated with 20 μg/mL Tuberculin (purified protein derivative, PPD) (Statens Serum Institut, Copenhagen, Denmark) for 16 h at 37°C with a humidified 5% CO_2_ atmosphere. GolgiStop (1.0 μg/mL) (BD Bioscience, San Jose, CA, USA) was added to cell suspension and incubated for extra 4 hrs. For the positive control, GolgiStop (1.0 μg/mL), phorbolmyristate acetate (PMA) (1.0 μg/mL), and ionomycin (10 μg/mL) (both from Sigma-Aldrich, St. Louis, MO, USA) were added to PBMCs and incubated for 4 h at 37°C in a humidified 5% CO_2_ atmosphere. Cells incubated in RPMI 1640 complete medium were treated as negative controls. A representative flow cytometric figure is shown in **Supplementary Figure 5**.

### Flow cytometry

After stimulation, cells were washed and incubated with fluorescence-conjugated monoclonal antibodies including Pacific Blue-conjugated mouse anti-human CD3, PE-Cy7-conjugated mouse anti-human CD8, FITC-conjugated mouse anti-human CD45RO and APC-cy7-conjugated mouse anti-human CD27 (all from BD Biosciences) for 40 min at 4°C. Cells were washed once with PBS containing 2% FCS, fixed and permeated using the Cytofix/Cytoperm^™^ Plus kit (BD Biosciences) according to the manufacturer’s instructions. Cells were incubated with PerCP-Cy5.5-conjugated mouse anti-human IFN-γ (BioLegend, San Diego, CA, USA), APC-conjugated mouse anti-human IL-2 (BD Biosciences) and PE-conjugated mouse anti-human IL-17A (BD Biosciences) for 45 min at 4°C. Cells were washed and resuspended in PBS, then acquired using a FACS X20 flow cytometer (BD Biosciences) within 2 h. Data were analyzed using FlowJo software 7.5 (FlOWJO LLC, Treestar, Ashland, OR, USA), and the doublet events were excluded by FSC-A/FSC-H gating.

### Interferon gamma release assay

PPD-specific IFN-γ release was determined by using an enzyme-linked immunospot (ELISpot) assay according to the manufacturer’s instructions (U-CyTech, Utrecht, Netherlands). Briefly, 96-well PVDF plates (Millipore, Bedford, MA, USA) were coated with anti-human IFN-γ coating antibody overnight at 4°C. The wells were blocked for 1 hr at 37°C, then 2.5 × 10^5^ PBMCs in 100 μL RPMI-1640 complete medium was inoculated into each well in the presence of PPD (20 μg/mL). RPMI-1640 complete medium served as a negative control and 2.5 μg/mL phytohemagglutinin (PHA) (Sigma-Aldrich) treatment as a positive control. After 20 h incubation at 37°C, the plates were incubated with a biotin-labeled detection antibody at 37°C for 1 hr and subsequently horseradish peroxidase (HRP)-conjugated streptavidin working solution for another 1 h. AEC substrate solution was added to each well for 30 min in the dark at room temperature. Color development was stopped by thoroughly rinsing both sides of the PVDF membrane with demineralized water. The plates were dried in the dark at room temperature. Spots were counted using a C.T.L. ImmunoSpot^®^ S6 Ultra Analyzer (Cellular Technology Limited, Shaker Heights, OH, USA). The number of BCG-specific IFN-γ-producing cells was calculated based on the spot-forming units (SFUs) per 2.5 × 10^5^ PBMCs after deducting the background SFUs of the paired negative control wells.

### Statistical analysis

All data are presented as the mean ± standard error of the mean (SEM). Data distributions and the test of normality were evaluated using the Shapiro-Wilk test. Statistical comparisons were performed using the statistical software package, SPSS 23.0 (SPSS, Chicago, IL, USA). The Mann-Whitney U test was used to evaluate two group differences. One-way analysis of variance (ANOVA) was used to assess differences between more than two groups. Statistical significance was defined as *P* < 0.05.

## Results

### Intravesical BCG instillations induce robust peripheral PPD-specific immune responses in bladder cancer patients

**Figure 1:**
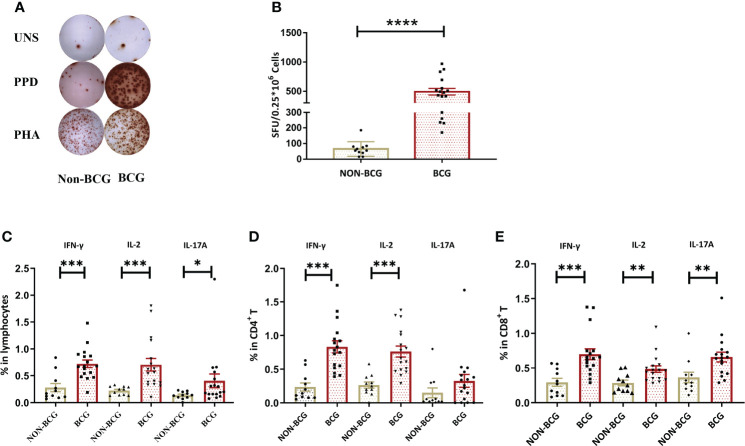
BCG treatment induces systemic antigen-specific cellular immune responses. **(A)** A representative result of the IFN-γ ELISpot assay. PBMCs from one patient receiving chemotherapy (NON-BCG) (left) and one patient receiving BCG instillation (BCG) (right) were stimulated with PPD and PHA (as the positive control), respectively. Cells without stimulation were used as a blank control (UNS). **(B)** Comparisons of the numbers of IFN-γ secreting cells [spot forming units (SFUs) per 250,000 PBMCs] between NON-BCG group (n = 11) and BCG group (N = 17) upon PPD stimulation. **(C–E)** PPD-specific cytokine production by total lymphocytes, CD4^+^ and CD8^+^ T cells. In total, 1.5 × 10^6^ PBMCs were stimulated by PPD. PPD-specific IFN-γ, IL-2, and IL-17A producing lymphocytes **(C)**, CD4^+^ **(D)**, or CD8^+^ **(E)** T cells were measured by flow cytometry. Data are represented as the mean ± SEM. The *P* values were calculated using the Mann-Whitney U test. ^*^ *P* < 0.05, ^**^ *P* < 0.01, ^***^ *P* < 0.001, ^****^ *P* < 0.0001.

To determine whether intravesical BCG instillation can induce systemic immune responses, whole blood from NMIBC patients post-TURBt surgery that either received intravesical chemotherapy (NON-BCG group) (n = 11) or BCG instillation at the maintenance phase (BCG group) (n = 17) was collected and PPD-specific IFN-γ release and cytokine-producing T cells (gating strategies and representative profiles are shown in **Supplementary Figure 4**) were compared between two groups. There was no difference in age, gender, or tumor grade between two groups (**Table 1**). There was a dramatic increase in PPD-specific IFN-γ producing cells in the periphery of BC patients from the BCG group, when compared to those in the NON-BCG group (*P* < 0.0001) (**Figures 1A, B**). Flow cytometric analysis revealed that higher percentages of IFN-γ (*P* < 0.001), IL-2 (*P* < 0.001) and IL-17A (*P* < 0.05) producing lymphocytes were present in the periphery of BCG group patients upon *in vitro* PPD stimulation, when compared with the NON-BCG group (**Figure 1C**). More specifically, both CD4^+^ (**Figure 1D**) and CD8^+^ T cells (**Figure 1E**) in the periphery of the BCG group produced IFN-γ, IL-2 and IL-17A to higher levels than those in the NON-BCG group, among which the frequencies of PPD-specific Th1-like cytokine producing cells were more dramatic. These results indicated that regional BCG instillation could trigger robust peripheral PPD-specific T cell responses in BC patients.

### Dynamics of peripheral PPD-specific cellular immune responses at the induction phase of intravesical BCG instillation

**Figure 2:**
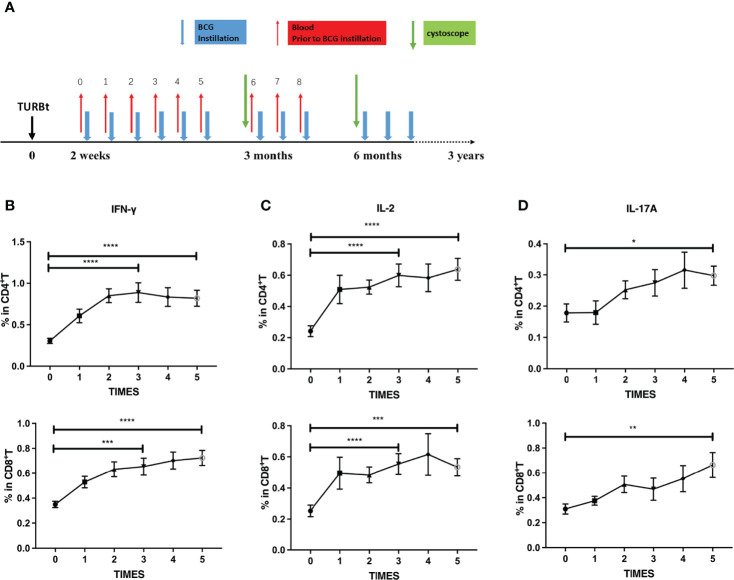
Dynamics of PPD-specific cytokine production in T cells at the induction phase. **(A)** Scheme of intravesical BCG instillation and blood sample collection in this study. Red arrows: blood sample collection (before each BCG instillation); Blue arrows: BCG instillation. **(B–D)** Dynamics of PPD-specific IFN-γ **(B)**, IL-2 **(C)** and IL-17A **(D)** producing CD4^+^ (up) or CD8^+^T (down) cells at the induction phase from BCG responding BC patients (N = 27). Data are represented as the mean ± SEM. The *P* values were calculated using analysis of variance. ^*^ *P* < 0.05, ^**^ *P* < 0.01, ^***^ *P* < 0.001, ^****^ *P* < 0.0001.

The aforementioned results indicated that long-term BCG instillations maintained high levels of peripheral PPD-specific cellular immune responses in NMIBC patients. We further investigated the dynamics of immune responses in BC patients receiving BCG instillation at the early stages of treatment with a serial collection of blood samples (**Figure 2A**). Interestingly, the frequencies of PPD-specific IFN-γ releasing CD4^+^ and CD8^+^ T cells increased (**Figure 2B**) after one dose BCG instillation, peaked after three BCG instillations, and remained at a high level. Consistently, IL-2 and IL-17A producing CD4^+^ or CD8^+^ T cells (**Figures 2C, D**) displayed similar kinetics in the periphery of BC patients receiving BCG instillation. By contrast, upon PMA/ionomycin stimulation, the numbers of T cells or cytokine-producing T cells were stable during treatment (**Supplementary Figures 1B–D**). Our results thus illustrated the rapid increase in peripheral BCG-specific immune responses during the early stage of BCG instillation. CD4^+^ and CD8^+^ T cells exhibited an enhanced capacity for PPD-specific Th1 and Th17-type cytokine production, further demonstrating the induction of systemic immune responses upon regional BCG treatment in BC patients.

### Peripheral PPD-specific cellular immune responses at the maintenance phase of intravesical BCG instillation

**Figure 3:**
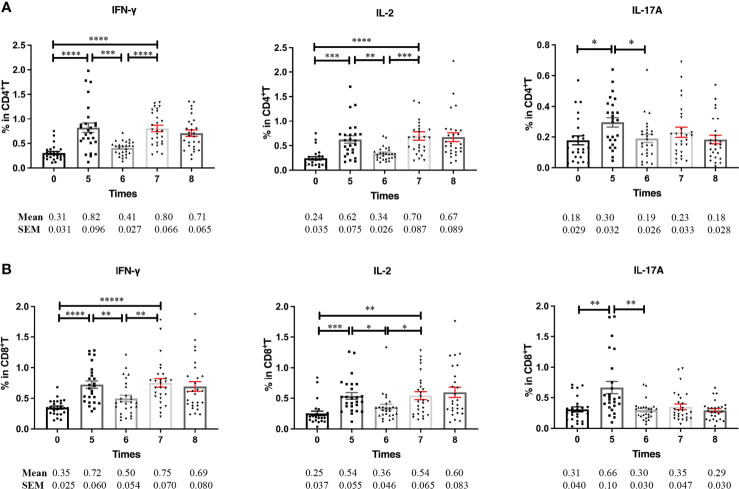
PPD-specific cytokine production in T cells at the maintenance phase. **(A, B)** Frequencies of PPD-specific IFN-γ, IL-2, and IL-17A releasing CD4^+^ **(A)** and CD8^+^ **(B)** T cells at the induction and maintenance phases. Blood samples were collected serially from BCG responding patients (n = 27) before each BCG instillation at the induction and maintenance phase. PBMCs were prepared and stimulated with PPD for further flow cytometric analyses. Data are represented as the mean ± SEM. *P* values were calculated using analysis of variance. ^*^ *P* < 0.05, ^**^ *P* < 0.01, ^***^ *P* < 0.001, ^****^ *P* < 0.0001.

After the induction phase, NMIBC patients continue to receive BCG instillation less frequently at 3-month intervals (defined as the maintenance phase). We characterized the PPD-specific immune profiles of the patients during the maintenance phases. Although PPD-specific Th1 and Th17-type cytokine production by CD4^+^ and CD8^+^ T cells was maintained at a high level during the induction phase, they decreased to pre-BCG instillation levels before receiving BCG instillation for the maintenance phase. After restarting BCG instillation, only PPD-specific Th1-type IFN-γ (**Figures 3A, B** **, left**) and IL-2 (**Figures 3A, B**, middle) secreting CD4^+^ and CD8^+^ T cells increased significantly, whereas PPD-specific IL-17A production by CD4^+^ T cells was not restored (**Figures 3A, B**, right). These results strongly indicated that repetitive BCG instillation induced PPD-specific Th1-type responses both in CD4^+^ and CD8^+^ T cells.

### High percentages of peripheral CD8^+^ T cells and the ratios of CD8^+^/CD4^+^ at baseline are associated with tumor recurrence-free prognosis

**Figure 4:**
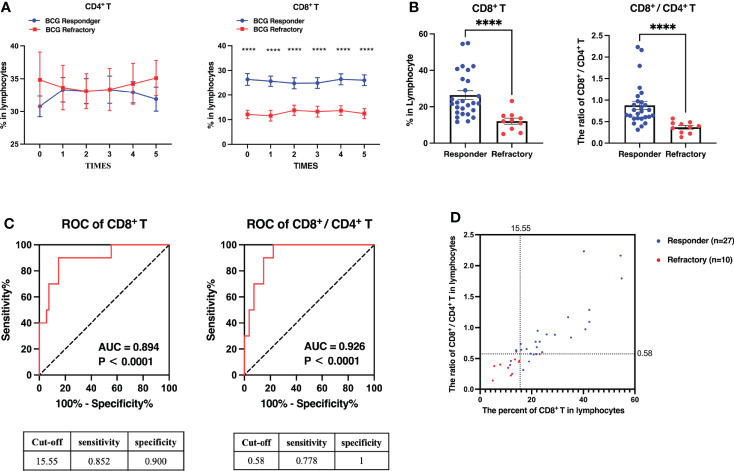
The percentages of CD8^+^ T cells and the ratio of CD8^+^/CD4^+^ at the baseline are more predicable for a better prognosis of BCG instillation. Patients were stratified into “BCG responder” (n = 27) and “BCG refractory” (n = 10) based on the clinical evaluation within 6 months, as described in the “Methods and Materials” section. **(A)** The percentages of CD4^+^ or CD8^+^ T cells among the total lymphocytes in the induction phase. **(B)** The percentages of CD8^+^ T cells and the ratios of CD8^+^/CD4^+^ before the BCG instillation. **(C)** ROC analysis of the percentages of CD8^+^ T cells and the ratio of CD8^+^/CD4^+^. **(D)** A scatter plot illustrated how recurrence-free patients and those with recurrence may be discriminated based on the percentages of CD8^+^ T cells and the ratio of CD8^+^/CD4^+^ cells before BCG instillation. Blue circles indicate BCG responder, red circles indicate BCG refractory. ****P < 0.0001.

Tumor recurrence within 6 months after BCG treatment is the key criteria for BCG refraction. Whether peripheral PPD-specific immune responses during the induction phase are predictive for tumor recurrence was investigated further. Among 37 NMIBC patients receiving BCG instillation, 27 patients were without tumor recurrence within 6 months (defined as “BCG responder”) and 10 patients underwent tumor relapse defined as “BCG refractory” (**Table 1**). However, there was an obvious increase in the percentages of peripheral CD8^+^ T cells in BCG responders, whereas no difference was detected in the frequency of CD4^+^ T cells between the two groups (**Figure 4A**). Individuals who were free from tumor recurrence after 6 months had higher proportions of CD8^+^ T cells and higher ratios of CD8^+^/CD4^+^ before the induction treatment (**Figure 4B**). The AUC values reached 0.926 for the ratio of CD8^+^/CD4^+^ (*P* = 0.000) and 0.894 for the frequency of CD8^+^ T cells (*P* = 0.000) (**Figure 4C**). When using cut-off values of 15.55 for the frequency of CD8^+^ T cells and 0.58 for the CD8^+^/CD4^+^ ratio, it was found that 25 of the 27 patients were recurrence-free survivors (92.6%) and nine of the 10 cases with recurrence (92.0%), with an overall correct classification for 34 of 37 patients (91.9%) (**Figure 4D**).

### Peripheral PPD-specific cellular immune response dynamics at the induction phase differ between BCG responder and BCG refractory groups

**Figure 5:**
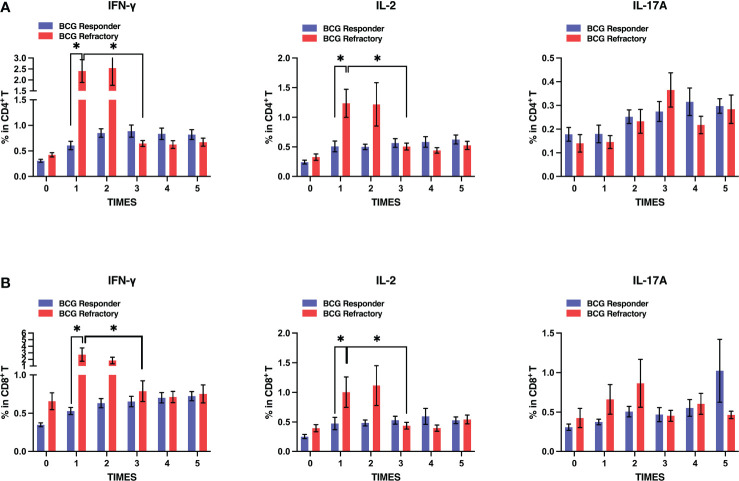
Dynamics of antigen-specific cytokines secretion by T cells in BCG responder and BCG refractory patients at the induction phase. Blood samples were collected and PBMCs were subjected to PPD stimulation and flow cytometric assays serially at the induction phase. **(A, B)** Frequencies of PPD-specific IFN-γ, IL-2, and IL-17A releasing CD4^+^ (up) or CD8^+^ T (down) cells in two groups. Data are represented as the mean ± SEM. *P* values were calculated using analysis of variance. *P < 0.05.

We further compared peripheral PPD-specific immune responses between the two groups. Unexpectedly, both CD4^+^ and CD8^+^ T cells exhibited significantly higher levels of PPD-specific IFN-γ and IL-2 production after the first and second BCG instillations in BCG refractory NMIBC patients when compared to BCG responders. However, this increase was transient. After the third instillation, the percentages of PPD-specific IFN-γ and IL-2 producing CD4^+^ and CD8^+^ T cells decreased to similar levels to BCG responders (**Figures 5A, B**). In contrast, IFN-γ producing CD4^+^ and CD8^+^ T cells in the BCG responders increased, then were constant after three BCG instillations, and maintained similar levels during the induction stage. Therefore, BCG refractory group patients exhibit more fluctuated PPD-specific cytokine productions by CD4^+^ or CD8^+^ T cells at the induction phage, whereas those in BCG responder group patients were more stable.

## Discussion

Despite clinical applications over the past 40 years, intravesical BCG instillation as a postsurgical adjuvant therapy shows 30%−50% unresponsiveness or relapses within 6 months (7) with no definitive intravesical salvage therapies. In this study, we defined the dynamics of PPD-specific cellular immune responses upon BCG instillation, which was intended to identify possible predictive indicators. Consistent with previous studies (16, 19), intravesical BCG instillation induced systemic PPD-specific cellular immune responses, which included increased numbers of PPD-specific Th1-type (IFN-γ and IL-2) and Th17 (IL-17A) cytokine producing T cells in the BCG group when compared to the NON-BCG group. When we investigated the dynamics of PPD-specific T cell responses at the induction phase, it was found that the percentages of peripheral CD4^+^ and CD8^+^ T cells were relatively stable (**Supplementary Figure 1A**) whereas PPD-specific IFN-γ, IL-2, and IL-17A secreting CD4^+^ and CD8^+^ T cells were augmented at early time points and remained at high levels. The rapid increase in BCG-induced cellular immunoreactivity after BCG instillation was consistent with several previous studies based on urine or in the serum. For example, higher amounts of IL-2 and IFN-γ were detected in the urine and serum after the fourth BCG instillation (22). It is worth noting that PPD-specific immune responses appear earlier in the Chinese population, and the immunoreactivity is stronger than that in Europeans (19, 22). This may be due to the fact that the majority of BC patients included in our study had a history of BCG vaccination. Therefore, BCG instillation led to repeat use of BCG and induced memory-like immune responses with rapidity and strength.

However, BCG-induced specific immunoreactivity was not durable after BCG intravesical instillation. Both the Th1 and Th17-type cytokine levels in CD4^+^ and CD8^+^ T cells at the beginning of the maintenance phase were decreased to the levels comparable to those at the beginning of the induction phase in our study. After BCG re-instillation, only Th1-type cytokine production by CD4^+^ and CD8^+^ T cells was restored to high levels, whereas Th17-type cytokine production remained at a low level. These results are partly consistent with a previous study that CD8^+^ and CD4^+^ T cells returned to the control levels by 3 weeks after the last BCG instillation (23). The difference in the restoration of PPD-specific Th1-type and Th17-type cytokine production after BCG re-instillation at the maintenance phase implied that BCG instillation was more likely to restore PPD-specific Th1-type cytokine production. In the context of tuberculosis field, BCG has been demonstrated to be able to induce antigen-specific Th1-type CD4^+^ T cells with production of IFN-γ, IL-2 and TNF-α, which is considered to exert the protection against *M.tb* infection (24, 25). The induction and maintenance of systemic PPD-specific immune responses by intravesical BCG instillation may help us to understand the mechanisms of BCG immunotherapy against NMIBC in addition to its local intervention. Furthermore, different cytokine dynamics of CD4^+^ and CD8^+^ T cells upon re-encountering BCG in adults provide useful clues for designing more efficient BCG strains to improve the efficacy of intravesical BCG instillation, potentially with the addition of extra signals.

A recent clinical trial has reported that shortened treatment at the maintenance was not as effective as standard protocol in BCG instillation treatment (4, 26). Because BCG instillation is a typical immunotherapy, assessing immune response might be more relevant to the prognostic values. Our results revealed that rather than PPD-specific immune responses, peripheral CD8^+^ T cell percentages and the ratios of CD8^+^/CD4^+^ exhibited more potential in predicting the efficacy of BCG instillation. This is also reasonable because PPD-specific immune responses are more likely to reflect the immunoreactivity to exogenous stimuli, whereas the infiltration of CD8^+^ T cells into the bladder is necessary for the anti-tumor activity of BCG instillation (27). It has been reported that a reduction in the frequency of CD4^+^ and CD8^+^ T cells in the peripheral blood after intravesical BCG instillation potentially indicated bladder tissue recruitment (28). Insufficient peripheral CD8^+^ T cells may reduce the recruitment of CD8^+^ T cells in the bladder with attenuated anti-tumor immunity after BCG instillation.

Our research unexpectedly found temporary increases in PPD-specific IFN-γ and IL-2 releasing CD4^+^ or CD8^+^ T cells in BCG refractory group when compared to the BCG responder group after the first and second BCG instillations. Furthermore, the dynamics of IFN-γ producing cells in CD3^-^ lymphocyte populations in the BCG responder and BCG relapsing groups were similar to those of CD4^+^ and CD8^+^ T cells (**Figure S2**). In addition, in the absence of CD3^-^ cells, the production of IFN-γ was impaired in both CD4^+^ or CD8^+^ T cells (**Figure S3**), which implied the involvement of innate cells in T cell activation. This was consistent with previous observation that BCG-responsive patients displayed lower expression of IFN-γ than BCG-refractory patients before the sixth instillation (29). Serum IFN-γ and IL-2R levels were higher in BCG refractory patients than in BCG-responsive patients. Although IL-2-mediated cancer immunotherapy has been used in the clinic for many years, high dose IL‐2 promotes the proliferation of cytotoxic effector T cells, as well as Treg cells, which can suppress the activity of effector T cells and thereby limit their antitumor efficacy (30, 31). IFN-γ is the central cytokine that mediates innate and adaptive immunity (32, 33). However, hyper-activity of IFN-γ has been reported to cause excessive tissue damage, necrosis, and inflammation and may contribute to disease pathology (34). Alberts et al. demonstrated that tumor progression was promoted upon IFN-γ administration (35) through a mechanism that involved upregulation of PD-L1 expression on tumor cells (36). Prolonged exposure to inflammatory cytokines has also been associated with tumor growth through promoting proliferation, angiogenesis, and DNA damage (37), and was associated with tumor relapse of the tumors during BCG treatment or carcinogenesis (38). Considering rapid decrease after third BCG instillation, it is more likely to indicate the contraction of BCG-induced T cell responses, which partially reflects the attenuation of T cell functionality. However, how fluctuated PPD-specific T cell functionality affects the outcomes of the reported BCG treatment needs further investigation.

## Conclusion

Collectively, our study revealed that BCG instillation rapidly induced BCG-specific T cell responses, and maintained it for a short duration. Repetitive BCG instillation at the maintenance phase restored partial BCG-specific T cell functionality. However, BCG-specific cellular responses were not associated with the reoccurrence of bladder cancer receiving BCG instillation. The percentages of CD8^+^ T cells and the ratio of CD8^+^/CD4^+^ at the baseline were more predictable, and were a better prognostic factor of response to BCG immunotherapy.

## Data availability statement

The raw data supporting the conclusions of this article will be made available by the authors, without undue reservation.

## Ethics statement

The studies involving human participants were reviewed and approved by The Ethics Committee of the Xinhua Hospital affiliated Shanghai Jiao Tong University School of Medicine. The patients/participants provided their written informed consent to participate in this study.

## Author contributions

HS, YWa, and SQ conceived the project and designed the experiments. HD and WX collected samples, performed the experiments, and analyzed the data. SQ, YD, CW, SZ, and YWu helped to collect the samples and clinical information. RS, and CY performed flow cytometry. YC, PJ, and SW provided the technical supports. HD, SQ, HS and YWa wrote the manuscript. DX revised the manuscript. All authors contributed to the article and approved the submitted version.

## Funding

This work was supported by grants from the National Key Research and Development Program of China (2021YFC2301500), the Chinese National Mega Science and Technology Program on Infectious Diseases (2018ZX10731301-001-004, 2018ZX10302301-002-002 and 2013ZX10003007-003-003), Shanghai Academic Research Leader Project (2018XD1403300), and the Science and Technology Commission of Shanghai Municipality (No. 21511902300).

## Acknowledgments

We thank all of the authors who participated in this project. The authors declare that they have no competing interests.

## Conflict of interest

The authors declare that the research was conducted in the absence of any commercial or financial relationships that could be construed as a potential conflict of interest.

## Publisher’s note

All claims expressed in this article are solely those of the authors and do not necessarily represent those of their affiliated organizations, or those of the publisher, the editors and the reviewers. Any product that may be evaluated in this article, or claim that may be made by its manufacturer, is not guaranteed or endorsed by the publisher.
