# Supplementary material for: Peripheral tuberculin purified protein derivative specific T cell immunoreactivity dynamics in non-muscle invasive bladder cancer patients receiving bacillus Calmette-Guerin instillation treatment
Source: Front Oncol. 2022 Oct 27;12:927410. doi: 10.3389/fonc.2022.927410 (PMC9646940; doi:10.3389/fonc.2022.927410)
Supplement: Supplementary file 1 [file DataSheet_1.docx]

**Supplementary Materials**

**Peripheral tuberculin purified protein derivative specific T cell immunoreactivity dynamics in non-muscle invasive bladder cancer patients receiving bacillus Calmette-Guerin instillation treatment**

Huangqi Duan^1^, Weimin Xia^1^, Yingying Chen^2^, Yu Ding^1^, Chen Wang^1^, Ruiming Sun^2^, Chengcheng Yao^2^, Shun Zhang^1^, Yu Wu^1^, Ping Ji^2^, Shujun Wang^2^, Subo Qian^1*^, Ying Wang^2*^, Haibo Shen^1*^

^1^Department of Urology, Xinhua Hospital, School of Medicine, Shanghai Jiao Tong University, Shanghai 200092, China

^2^Shanghai Institute of Immunology, Department of Immunology and Microbiology, Shanghai Jiao Tong University School of Medicine, Shanghai 200025, China

Supplementary figures


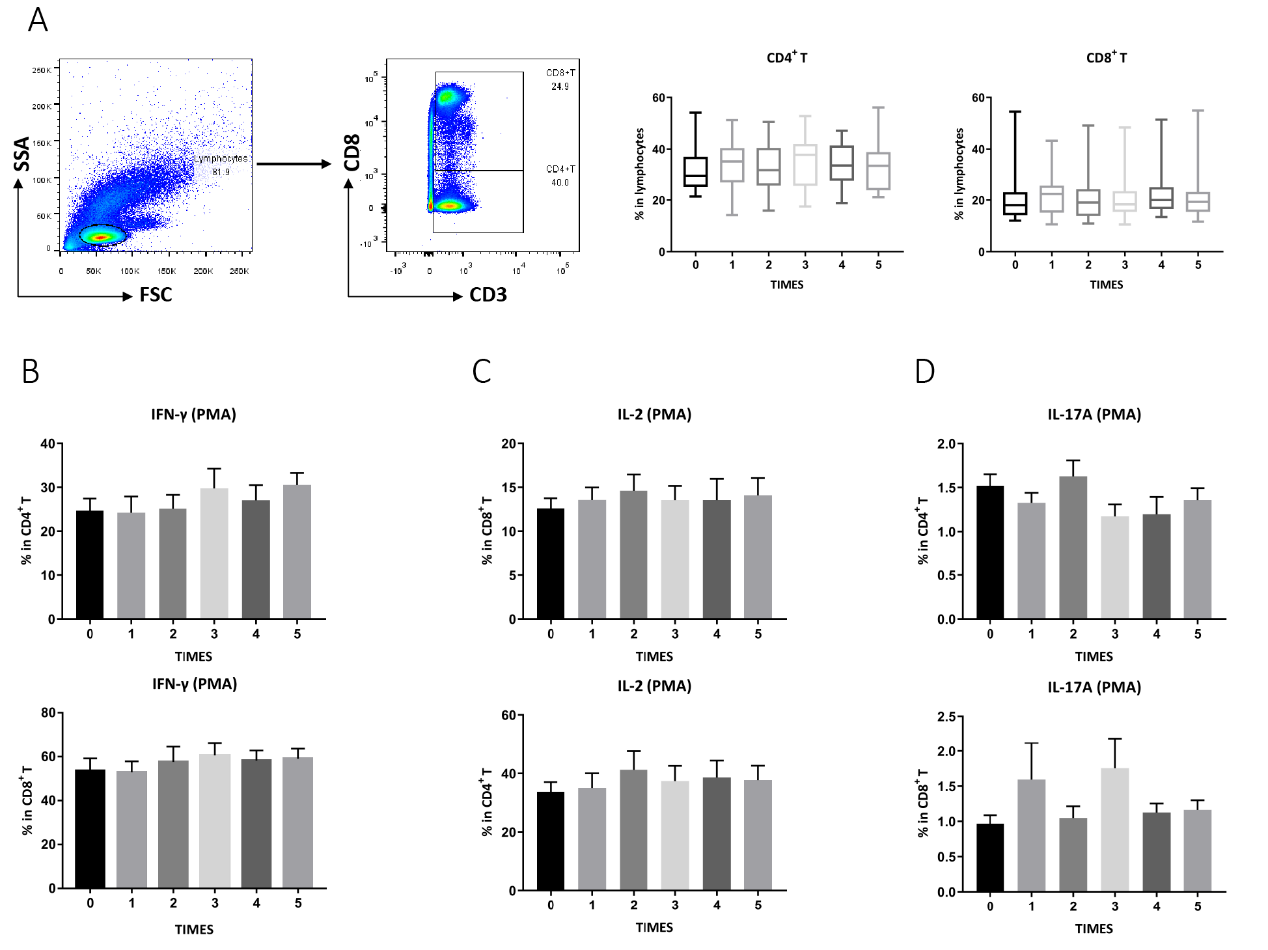


Supplementary figure 1: (A) Gating strategies of CD4^+^ and CD8^+^T cells in the lymphocytes and the percentages of CD4^+^ T and CD8^+^ T cells in the lymphocytes from BC patients at the induction phase. (B-D) Frequencies of IFN-γ (B), IL-2 (C) and IL-17A (D) secreting CD4^+^ (up) and CD8^+^ (down) T cells upon PMA/Ionomycin stimulation.


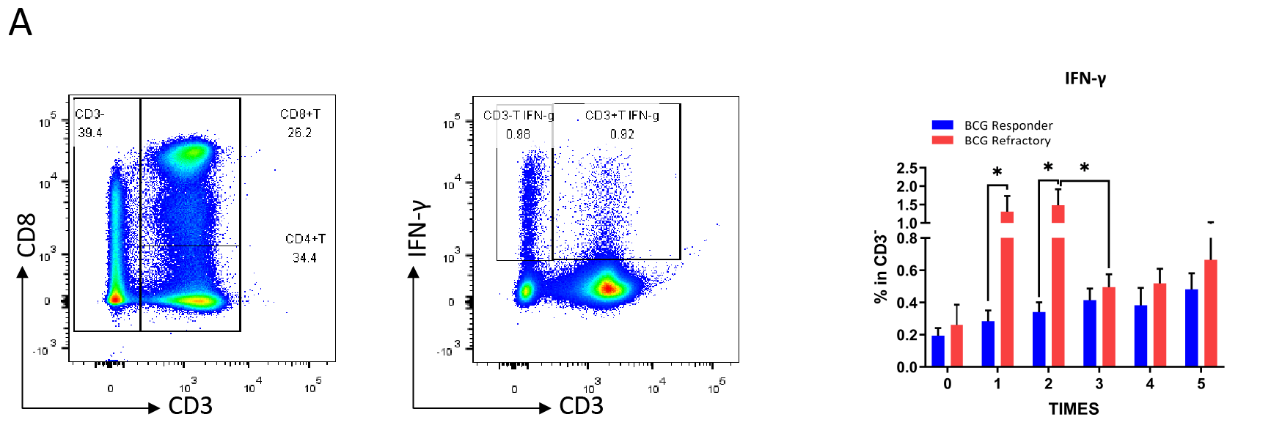


**Supplementary figure 2** Dynamics of PPD-specific IFN-γ producing CD3^-^ cells frequencies in BCG responder and BCG refractory patients at the induction stage**.**


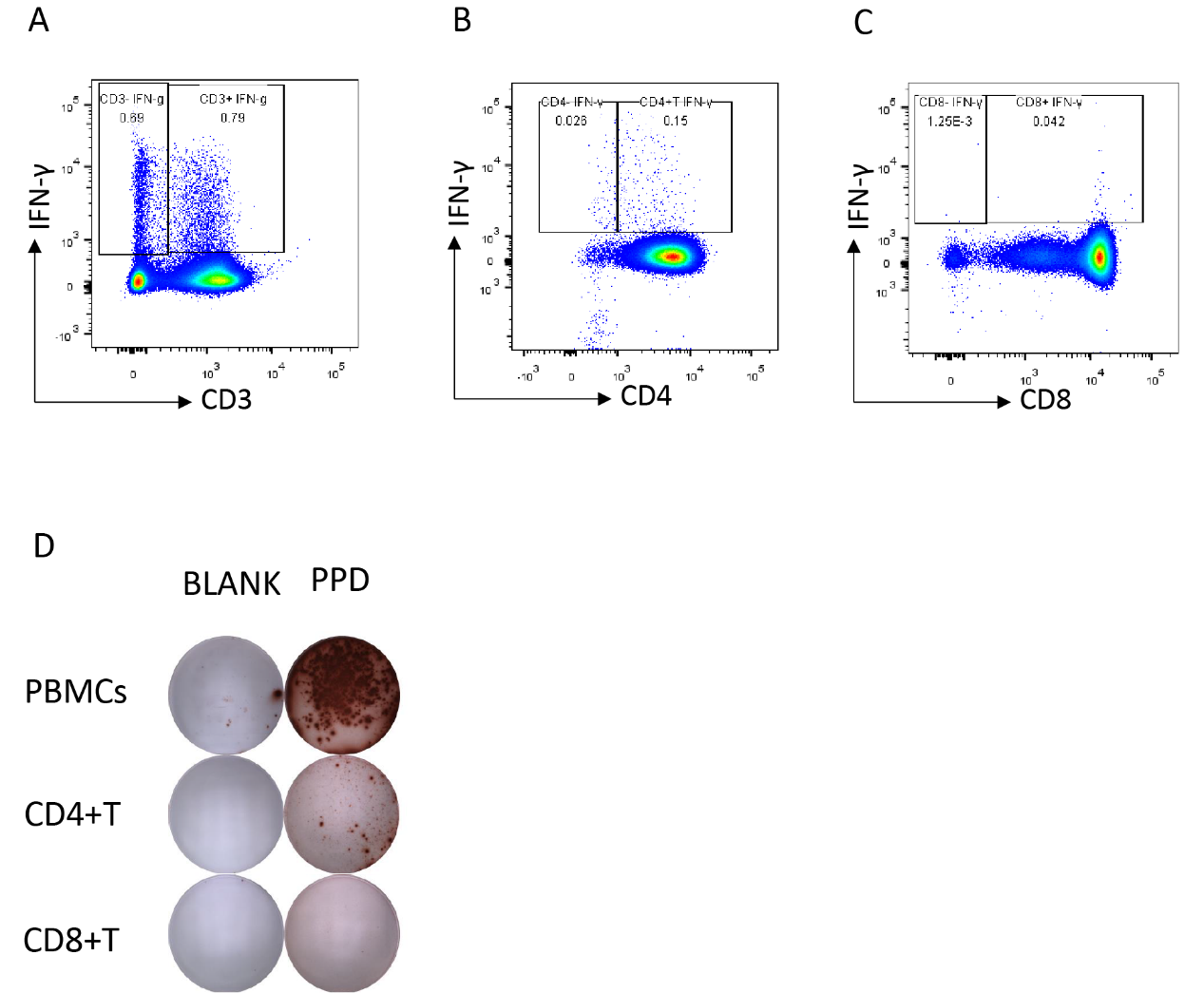


**Supplementary figure 3:** Peripheral blood mononuclear cells (PBMCs), purified CD4^+^ and CD8^+^T cells from one bladder cancer patients were stimulated with PPD. The frequency of IFN-γ releasing cells in PBMCs (A), CD4^+^ (B) and CD8^+^ T (C) were detected by flow cytometry and the ELISpot assay (D).


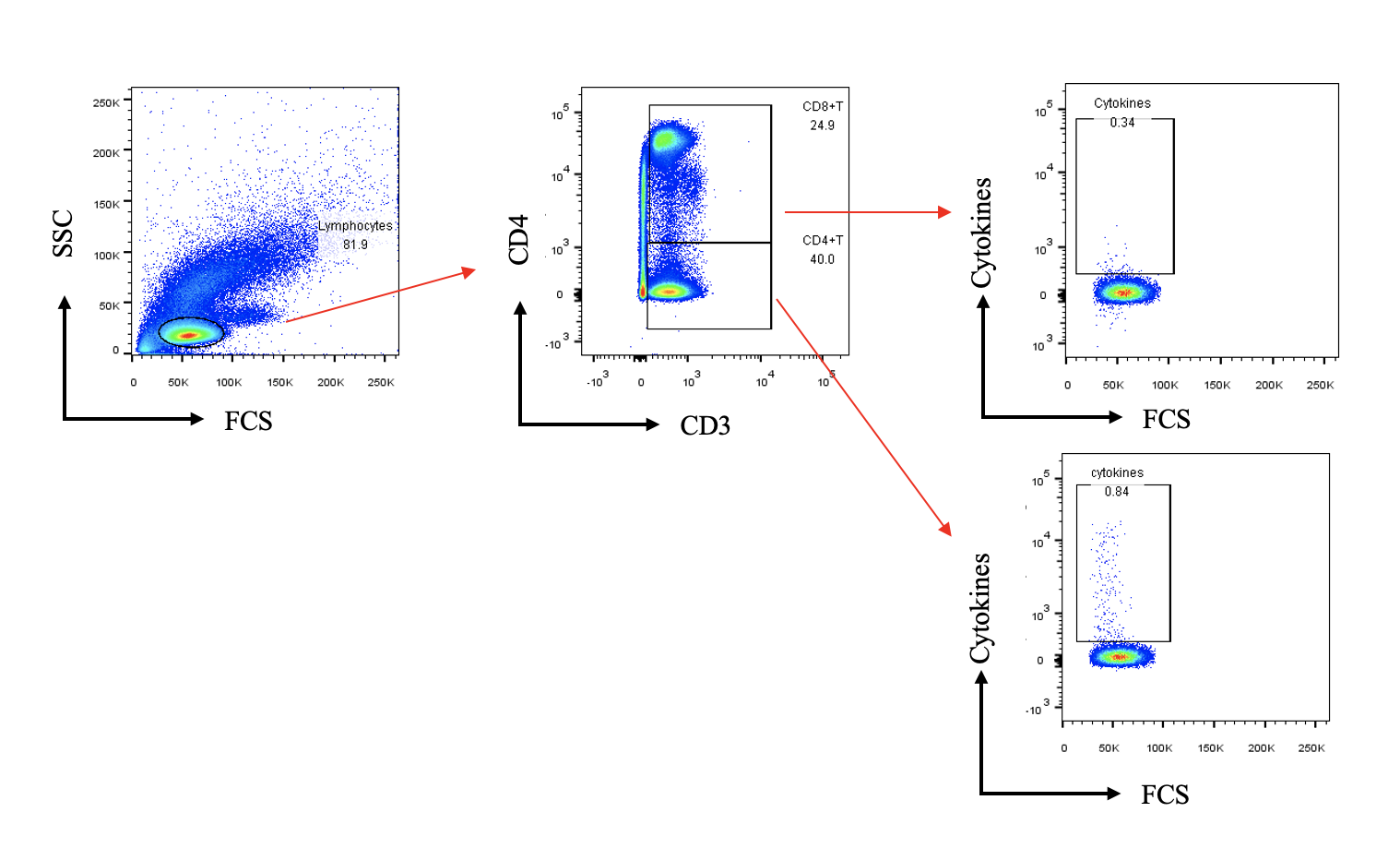


Supplementary figure 4: Gating strategies of flow cytometry.


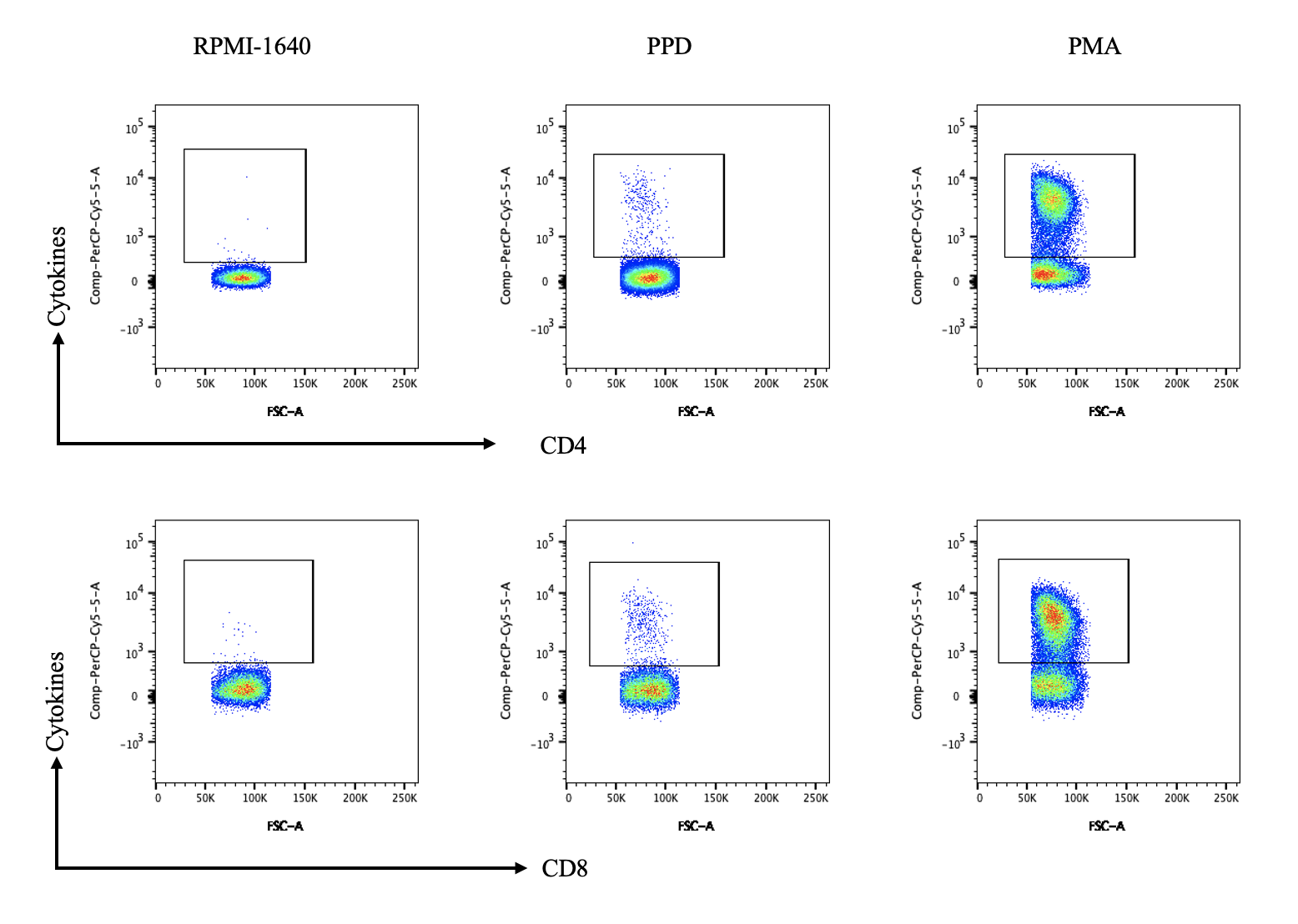


Supplementary figure 5: Representative flow cytometric figure. PBMCs from one patient receiving BCG instillation were stimulated with RPMI-1640 (as a negative control), PPD (middle) and PMA (as the positive control, right), respectively.
